# CucumberAI: Cucumber Fruit Morphology Identification System Based on Artificial Intelligence

**DOI:** 10.34133/plantphenomics.0193

**Published:** 2024-06-27

**Authors:** Wei Xue, Haifeng Ding, Tao Jin, Jialing Meng, Shiyou Wang, Zuo Liu, Xiupeng Ma, Ji Li

**Affiliations:** ^1^ College of Artificial Intelligence, Nanjing Agricultural University, Nanjing 210095, China.; ^2^ State Key Laboratory of Crop Genetics and Germplasm Enhancement, College of Horticulture, Nanjing Agricultural University, No. 1 Weigang, Nanjing 210095, China.; ^3^ College of Foreign Studies, Nanjing Agricultural University, Nanjing 210095, China.

## Abstract

Cucumber is an important vegetable crop that has high nutritional and economic value and is thus favored by consumers worldwide. Exploring an accurate and fast technique for measuring the morphological traits of cucumber fruit could be helpful for improving its breeding efficiency and further refining the development models for pepo fruits. At present, several sets of measurement schemes and standards have been proposed and applied for the characterization of cucumber fruits; however, these manual methods are time-consuming and inefficient. Therefore, in this paper, we propose a cucumber fruit morphological trait identification framework and software called CucumberAI, which combines image processing techniques with deep learning models to efficiently identify up to 51 cucumber features, including 32 newly defined parameters. The proposed tool introduces an algorithm for performing cucumber contour extraction and fruit segmentation based on image processing techniques. The identification framework comprises 6 deep learning models that combine fruit feature recognition rules with MobileNetV2 to construct a decision tree for fruit shape recognition. Additionally, the framework employs U-Net segmentation models for fruit stripe and endocarp segmentation, a MobileNetV2 model for carpel classification, a ResNet50 model for stripe classification and a YOLOv5 model for tumor identification. The relationships between the image-based manual and algorithmic traits are highly correlated, and validation tests were conducted to perform correlation analyses of fruit surface smoothness and roughness, and a fruit appearance cluster analysis was also performed. In brief, CucumberAI offers an efficient approach for extracting and analyzing cucumber phenotypes and provides valuable information for future cucumber genetic improvements.

## Introduction

Cucumber (*Cucumis sativus* L.) is an important vegetable crop that is widely cultivated worldwide. In Cucurbitaceae crops, cucumber is also an important model plant that exhibits a rich diversity in appearance. The different cucumber appearances can influence yield, marketability, processing adaptability, and even product safety [1,2]. For example, longer cucumbers have greater single weights but are prone to being malformed, sometimes having curved shapes. Fewer cucumber fruit tumors could be favored by consumers, while the processed varieties need more fruit tumors to improve processing adaptability [3]. Dense cucumber fruit thorns can confer some insect resistance, but they greatly reduce the quality of the fruit’s appearance and introduce some hidden dangers from pesticide residues [4,5]. Currently, fruit shape breeding has become an important modern cucumber improvement task.

In recent years, many breakthroughs have been made in understanding the inheritance basis and regulatory mechanism of cucumber fruit development [6–11]. However, most of the appearance characteristics of cucumbers are complex quantitative traits; due to the limitations of the traditional identification methods in terms of resolution, accuracy, and measurement efficiency, most of the genetic loci have not been detected thus far. Therefore, exploring a high-precision, real-time, and fast identification and measurement technology for cucumber fruit appearance is highly important for cucumber fruit improvement efficacies and refining the development of the pepo fruit model. Image recognition technology is an important field of artificial intelligence (AI) that has the advantages of fast speed, real-time operation, nondestructiveness, multiple features, and high-precision classification. It has been widely applied in agricultural resource identification and crop phenotypic omics research [12–14].

Currently, research on cucumber phenotypes primarily focuses on the extraction of cucumber morphological parameters [15], cucumber grading [16,17], image segmentation and cucumber growth environment classification, cucumber disease detection, etc. These studies predominantly involve the acquisition of simple cucumber parameters, such as length, width, and curvature; cucumber grading based on morphological data; and overall or local segmentation and classification of fruits and backgrounds. The lack of comprehensive research on automatic cucumber trait identification makes it difficult to meet the requirements of genetic breeding analysis. The key factor for extracting cucumber trait features is to achieve precise identification of the various parts of a cucumber fruit, including the stem, tip, stalk and body, stripes and tumors in the surface image, as well as the sarcocarp and endocarp in the cross-sectional image. Subsequently, various cucumber trait features are extracted. There are several critical challenges for cucumber trait identification: (a) defining scientifically comprehensive cucumber feature parameters, (b) extracting the cucumber fruit skeleton and segmenting the fruit based on its morphological characteristics, (c) segmenting the surface stripes and classifying them based on their features, (d) locating and identifying surface tumors, and (e) segmenting the sarcocarp and endocarp in the cross-sectional image and designing a method for carpel counting.

The features of cucumber fruit are divided into primary characteristics and advanced characteristics. The primary features include simple shape features such as length, width, and curvature. The advanced features include cucumber shape, stripe, fruit tumor density, endocarp segmentation, and carpel counting, some of which were identified for the first time based on deep learning models. Cucumber trait identification involves extracting the primary characteristic features using traditional digital image processing techniques and extracting the advanced characteristic features using deep learning methods. In the agricultural field, digital image processing techniques are widely used [18]. Morphology is one of the most mature techniques used for image processing and is mainly used for extracting image components, such as boundaries and connected regions [19]. Advanced characteristic feature descriptors in image processing are used to extract, encode, and classify an object’s texture, shape and other features [20]. Using these techniques, we can identify the contours, different parts of a fruit, and the morphological features. With the emergence of convolutional neural networks, deep learning techniques with excellent feature extraction and generalization abilities have gradually been used for segmentation, identification, and classification of rice, wheat, fruits, and flowers [21–29]. The speed and accuracy of these methods are generally greater than those of the traditional segmentation and object identification algorithms [30]. For example, DeepLab-ResNet [31] has been used for the segmentation and identification of apple, peach, and pear fruits in various environments. The same-colored fruit segmentation model based on Polar-Net [32] improves the harvesting efficiency of same-colored fruits in complex environments. The YOLOv5-LiNet and YOLOv5-4D fruit and tomato instance identification models [33,34] are used for enhancing fruit identification. A deep learning counting method [35] based on drone images transforms the field counting of rapeseed clusters into a density estimation problem and represents a novel approach for carpel counting. Therefore, deep learning models enable the extraction of the advanced characteristic features of cucumber, such as stripe type, tumor density, and carpel number.

To achieve automatic cucumber trait identification, we develop a new application called CucumberAI, which can extract 51 cucumber features from cucumber images. Among them, 32 features are adopted for the first time. CucumberAI introduces a pipeline for extracting morphological features of the primary characteristics based on digital image technology. Cucumber trait identification rules are combined with AI classification models for fruit trait identification. In addition, U-Net semantic segmentation models [36] are used for stripe and endocarp segmentation, an improved MobileNetV2 model [37] is used for carpel classification, a ResNet50 model [38] is used for stripe classification, and a YOLOv5 model is used for tumor identification. CucumberAI can accurately identify 11 traditional cucumber fruit shapes and 27 newly defined characteristic parameters to provide a more accurate and comprehensive description of cucumber fruits. This method of constructing data-based fruit models is of considerable importance for high-throughput mining of the genetic loci for fruit shape based on the genome-wide association study strategy. We used a large number of cucumber images to evaluate the accuracy of the identification parameters and their relationships with manual measurements. Compared to previous methods, this work represents a substantial advancement in trait diversity, identification accuracy, and software functionality.

## Materials and Methods

### Plant material and growing conditions

Approximately 229 cucumber high-generation inbred lines were used in the present study [39]. Plants were grown in plastic greenhouses at the Baima Scientific Research Experimental Base of Nanjing Agricultural University in Nanjing, Jiangsu Province, China. A randomized block design experiment was conducted. Ten individuals of each inbred line were sown within 0.3 × 0.3-m space per plant by routine field management. For each accession, 10 to 20 fruits (8 to 12 d after flowering) were harvested for data collection from the 15th to 20th nodes of the plants.

### Image acquisition

The fruits were brought into the photography room immediately after being picked, and photos were collected by using a Nikon d750 camera with a Nikon 24-70 mm/1: 28 G ED lens. The distance between the lens and the subject was 100 cm. To capture cucumber appearance images, fruits were placed flat on a black curtain with a 100-cm ruler and standard RGB cards. To capture fruit flesh images, the fruit was cut into slices of 1-cm thickness, and the pieces from the middle of the fruit body were selected and placed flat on a black curtain with a ruler and standard color cards. The images were in JPG format with a resolution of 4,016 × 6,016 pixels. A total of 1,811 frontal photographs of fruits and 1,369 cross-sectional photographs of fruits were collected.

### Trait indicators

The cucumber fruit trait indicators listed in Table 1 were measured by using traditional measurement and image recognition methods. The fruit appearance-related trait indicators were divided into 3 groups: fruit shape indicators, fruit surface indicators, and fruit flesh indicators. Five agronomic trait indicators, namely, fruit shape category, fruit neck shape, fruit tip shape, fruit rib depth, and fruit tumor size, were measured by the traditional grading method described in [40]. Using the traditional methods, each fruit is measured 3 times, and the average value is taken. In the image recognition method, secondary parameters are set to describe complex traits; for instance, the fruit contour shape is decomposed into 28 secondary parameters, the fruit tip shape is decomposed into 3 secondary parameters, and the fruit neck shape is decomposed into 3 secondary parameters.

**Table 1.** List of trait indicators

Traits	Secondary traits	Parameter name (unit)	Description and formula
Features of the fruit shape	/	*Fd* (cm)	Fruit diameter
		*Fl* (cm)	Fruit length
		*Ln* (cm)	Length of fruit neck
		*Lb* (cm)	Length of fruit body
		*Lt* (cm)	Length of fruit tip
		*Cf*	Category of fruit shape
		*Gn*	Grade of fruit neck
		*Gt*	Grade of fruit tip
	Shape of fruit contour	*S* (cm^2^)	Area of fruit contour
		*Lc* (cm)	Perimeter of fruit contour
		*V* (cm^3^)	Volume of fruit, *V* = *pi* * (*Fd*/2)^2^ * *Fl*
		*Ll* (cm)	Long axis length of fit ellipse
		*Ls* (cm)	Short axis length of fit ellipse
		*Lout* (cm)	Length of circumscribed rectangle
		*Wout* (cm)	Width of circumscribed rectangle
		*Dr*	Ratio of fruit contour to circumscribed rectangle, *Dr* = *S*/(Lout*Wout)
		*Hr_f_*	Ratio of fruit contour to convex-hull, *Hr_f_* = *S*/Convex-hull area of fruit
		*Cr_f_*	Ratio of fruit contour to circumferential circle, *Cr_f_* = *S*/Circumferential circle area of fruit
		*Er_f_*	Ellipse filling rate of fruit, *Ef* = *pi* *Ll*Ls/*S*
		*Cf_f_*	Ratio of fruit contour to inscribed circle, *Cf_f_* = Maximum inscribed circle area/*S*
		*Rl_f_*	Circularity of fruit, *Rl_f_* = 4*pi*S/*Lc*^2^
		*Cv*	Curvature
		*Nr*	Ratio of fruit neck, *Hr* = *Ln*/*Lb*
		*Tr*	Ratio of fruit tip, *Tr* = *Lt*/*Lb*
		*Ar*	Index of fruit body, *Ar* = *Lout*/*Wout*
		*Dr_f_*	Fluctuation ratio of fruit body diameter, *Dr_f_* = Standard deviation of fruit body diameter/Mean value of fruit body diameter
		*Dr_n_*	Ratio of fruit neck diameter to fruit body diameter, *Dr_n_* = The widest diameter of fruit neck/middle diameter of fruit body
		*Dr_t_*	Ratio of fruit tip diameter to fruit body diameter, *Dr_n_* = The widest diameter of fruit tip/middle diameter of fruit body
	Shape of the fruit neck	*Na* (°)	Angle of fruit neck, *Na* = The angle between the widest points and endpoint of the fruit neck
		*Ni*	Index of fruit neck, *Ni* = *Ln*/the widest diameter of fruit neck
		*Np*	Proportion of fruit neck, *Np* = The area between the widest points and endpoint of the fruit neck/area of fruit neck
	Shape of the fruit tip	*Ta* (°)	Angle of fruit tip, *Ta* = The angle between the widest points and endpoint of the fruit tip
		*Ti*	Index of fruit Tip, *Ti* = *Lt*/the widest diameter of fruit tip
		*Tp*	Proportion of fruit Tip, *Tp* = The area between the widest points and endpoint of the fruit tip/area of fruit neck
Features of the fruit surface	/	*Gfr*	Grade of fruit ribs
		*Gtu*	Grade of tumor
		*Td*	Density of tumor
		*Sr*	Proportion of stripe, *Sr* = Area of stripe/*S*
		*St*	Types of stripes
	Smoothness	*Hr_s_*	Ratio of sarcocarp contour to convex-hull, *Hr_s_* = *S*/area of convex-hull
		*Cr_s_*	Ratio of sarcocarp contour to circumferential circle, *Cr_s_* = *S*/area of circumferential circle
		*Rl_s_*	Circularity of sarcocarp contour, *Rl_s_* = 4*pi*S/*Lc*^2^
		*Cf_s_*	Ratio of sarcocarp to inscribed circle, *Cf_s_* = Area of inscribed circle/*S*
		*Cdv*	Variance of distance from centroid to edge
Features of the fruit flesh	/	*Tm* (cm)	Thickness of mesocarp, Tm=Ss+Se/pi−Re
		*Re* (cm)	Radius of endocarp $\mathrm{Pct}=\sqrt{\mathrm{Se}/\mathrm{pi}}$
		*Ptr*	Ratio of mesocarp, *Ptr* = *Tm*/*Re*
		*Ss* (cm^2^)	Area of sarcocarp
		*Se* (cm^2^)	Area of endocarp
		*Pe*	Proportion of endocarp, *Pe* = *Se*/*Ss*
		*Nc*	Number of carpels

### Establishment of CucumberAI

**Fig. 1.**
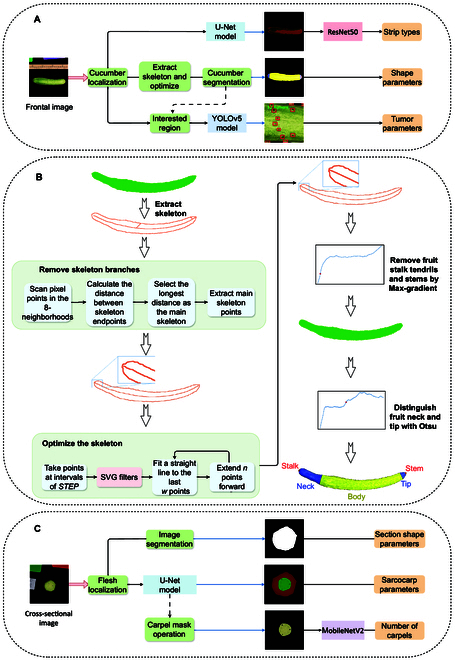
Pipeline overview of CucumberAI. (A) Detection of cucumber shape parameters, stripe parameters, and fruit tumor density through image morphology processing, deep learning segmentation, and object detection models. (B) Process of cucumber segmentation through skeleton extraction and optimization. (C) Processes of the shape parameters, sarcocarp (including the mesocarp in red and the endocarp in green) parameters, and carpel number detection. SVG, Savitzky–Golay.

**Fig. 2.**
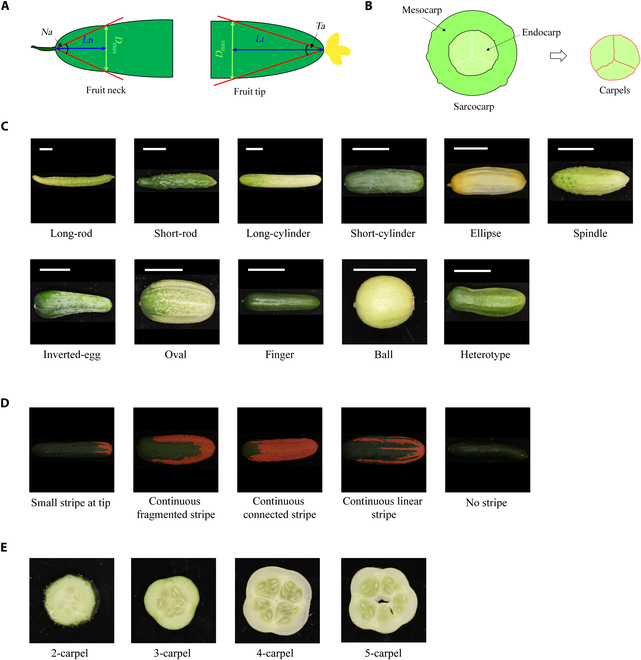
The appearance of cucumbers. (A) Exploded view of the neck and tip. *D_max_* indicates the widest diameter of the fruit neck or tip. (B) Schematic diagram of the sarcocarp. The carpels are outlined with red lines. (C) Fruit shape categories. (D) Types of stripes marked in red. (E) Carpel classification.

Here, we present a tool for the automatic identification of cucumber traits that is designed to work across cucumber varieties and different imaging conditions. The algorithms used in the proposed tool are implemented based on machine vision and deep learning models. The pipeline starts with an extraction of cucumber features using frontal and cross-sectional images. (a) The frontal image of the cucumber fruit is identified to extract its shape features, stripe features, and tumor density (Fig. 1A). First, the shape parameters are extracted. The supergreen algorithm [41] converts the frontal image into a supergreen grayscale image to filter out nongreen objects, thereby obtaining the cucumber fruit and green color card as a scale reference. Subsequently, an opening operation and multiple rounds of erosion remove small objects that affect the contour extraction in the image. We select 2 contours with the maximum areas from all the contours. Among them, the one with the highest degree of rectangularity is the reference object. Based on the actual and pixel lengths of the color-card contour, we calculate the unit length (pixels per centimeter) of the image. Moreover, the contour with the lowest degree of rectangularity is the cucumber contour, which is filled with white to generate a black-and-white binary image. Based on the cucumber contour, we calculate the *S*, *Lc*, *V*, *Lout*, *Wout*, *Ls*, *Ll*, *Hr_f_*, *Cr_f_*, etc. Furthermore, based on the Zhang–Suen thinning algorithm [42] and a series of steps, the binary image skeleton of cucumber is extracted and optimized (Fig. 1B). Then, the transverse diameter of the fruit is calculated based on the skeleton and contour information, and the fruit segmentation is implemented based on the trend exhibited by the changes in the fruit’s transverse diameter. The fruit is divided into 5 segments, i.e., the stalk, neck, body, tip, and stem (Figs. 1B and 2A). Based on the optimized skeleton, parameters such as *Fl* and *Cv* can be obtained. Thus, the other cucumber shape parameters listed in Table 1 are calculated. Second, the surface stripe is segmented based on the U-Net model, the stripe image is classified based on the ResNet50 model (Fig. 2D), and the proportion of stripe *Sr* is calculated using the area of fruit contour *S*. Finally, the square area with the midpoint of the skeleton as the center, and the side length as the lateral diameter at the midpoint of the skeleton, is considered the region of interest. The number of fruit tumors is detected in the region of interest using the YOLOv5 model, and the density of fruit tumors *Td* is calculated according to the area of interest (Fig. 1A). (b) The cross-sectional image of the fruit sarcocarp is used to determine the geometric and sarcocarp parameters (Fig. 1C), such as the thickness of the mesocarp (*Tm*) and the number of carpels (*Nc*). The supergreen algorithm is used to convert the cross-sectional image into a supergreen grayscale image. An opening operation and multiple rounds of erosion are used to remove noise that affects the contour extraction. We select the 2 contours with the largest areas and calculate their degrees of circularity. The area with a higher degree of circularity corresponds to the sarcocarp cross-section and is filled to generate a black-and-white binary image. Based on the binary image and contour information, geometrical parameters, such as the cross-sectional area of the sarcocarp *Ss*, circularity *Rl_s_*, circle filling rate *Cf_s_* and variance of the centroid-to-contour distance *Cdv*, are used to describe the smoothness of the fruit surface. Based on the U-Net model, the mesocarp and endocarp regions are segmented to obtain their corresponding areas (Fig. 2B). Assuming that the endocarp and sarcocarp are circular, we calculate their radius and difference as the thickness of the mesocarp *Tm.* In addition, *Pcr* and *Ptr* can also be calculated. The MobileNetV2 model extracts depth features from the endocarp region images and classifies the carpel into 4 categories, namely, 2-carpel, 3-carpel, 4-carpel, or 5-carpel (Fig. 2E), to obtain the number of carpels *Nc*. Finally, all the trait parameters are exported into an XLS file, and multiple identification result images are generated.

### Skeleton extraction and optimization

This process uses the Zhang–Suen thinning algorithm to extract and optimize the binary image skeleton of the cucumber plants. First, we scan the 8 neighborhoods of all the pixels and consider the pixel with only 1 white neighborhood as the skeleton endpoint. Then, we calculate the distance between each pair of endpoints. We denote the pair of endpoints with the longest distance as the skeleton trunk endpoints and the path connecting this pair of endpoints as the skeleton trunk, which is retained in the binary image. Other endpoints are branches and are removed from the skeleton. Next, we select points on the skeleton with a step of *STEP* and use a Savitzky–Golay filter [43] with a window size of *w* to filter points to generate a smooth skeleton. After that, the skeleton extracted above is incomplete at both ends of the fruit, and it needs to be extended to the fruit contour. At each end of the skeleton, we select *e* points for polynomial curve fitting. We use the fitted polynomial to calculate *n* points backward. This process is repeated until the skeleton intersects with the fruit contour. Finally, we remove the out-of-contour portion of the skeleton to generate the optimized skeleton.

### Cucumber segmentation

Based on the skeleton and contour, cucumber segmentation divides the fruit into 5 segments (Fig. 1B): the stalk, neck, body, tip, and stem. To recognize the tip and neck (Fig. 2A), we split the contour and skeleton into left and right parts from the middle. For the left fruit neck, we calculate the minimum distance from the skeleton point to the contour in order from left to right and use Otsu to divide the distance list into 2 categories. We consider the skeleton point corresponding to the maximum distance between the 2 categories as the neck boundary. The same method is applied to locate the fruit tip. To identify the left-side stalk, we draw perpendicular lines from all the skeleton points to the contour to obtain the distance between the 2 intersection points on the contour and obtain all the transverse diameters at the fruit tip. Subsequently, we calculate the gradient of the transverse diameter curve. We identify the skeleton point with the maximum gradient value as the stalk boundary. The same method is applied to locate the fruit stem. Finally, we fill the stalk, tip, neck, and stem of the fruit with colors to generate a segmentation image.

### Shape classification

The shape of a cucumber fruit can be divided into 11 types (Fig. 2C): long-rod, short-rod, long-cylinder, short-cylinder, ellipse, spindle, inverted-egg, oval, finger, ball, and heterotype. The method for classifying cucumber shapes into the above types combines the empirical values of the cucumber trait parameters with an AI classification model. The method comprises the following steps. The fruits of ball, oval, and finger types exhibit considerably different circle filling rates. Hence, if the circle filling rate of a cucumber fruit is greater than *θ*_1_, it can be classified as a ball type. For nonball samples with a length less than *L*, if the circle filling rate is greater than *θ*_2_, they are classified as the oval type; otherwise, they are classified as the finger type. Furthermore, the long- and short-rod types are combined into the “rod” type, while the long- and short-cylinder types are combined into the “cylinder” type. Hence, there are 6 fruit shapes, i.e., rod, cylinder, ellipse, heterotype, spindle, and inverted egg. The MobileNetV2 classification model is trained with standard samples of these 6 types. Finally, the “rod” and “cylinder” types are further distinguished based on fruit length. If the length is greater than *T*, the fruit is classified as a long-rod or long-cylinder fruit; otherwise, it is classified as a short-rod or short-cylinder fruit. Hence, the fruits can be classified into 11 types.

## Results

### Network training

Network training involves the use of the U-Net semantic segmentation model for stripe segmentation and fruit flesh endocarp segmentation, the ResNet50 stripe classification model, MobileNetv2 for carpel classification (CarpelMobileNet), MobileNetv2 for shape classification (CarpelMobileNet), and the YOLOv5 object detection model. The training losses of these models are recorded, and their evaluation results were compared (Fig. 2). The experiment was implemented on a computer running Microsoft Windows 10 equipped with an Intel Core E5-2650 v4 central processing unit (frequency of 2.2 GHz) accelerated by 2 NVIDIA GeForce GTX 1080Ti graphics processing units. The model code was written in Python 3.6 and powered by PyTorch 1.2.0 and Torchvision 0.4.0. During training, 80% of the dataset was used as the training sample, and 20% was used as the validation sample. We provide a set of reference values for fruit segmentation, namely, *STEP* = 5, *w* = 3, *e* = 5, *k* = 1, and *n* = 3, and classification, namely, *θ*_1_ = 0.75, *θ*_2_ = 0.4, *L* = 10 cm, and *T* = 20 cm.

**Fig. 3.**
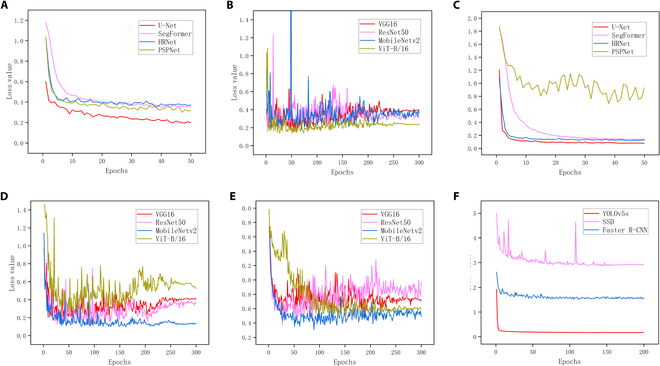
The losses of the models. (A) Fruit stripe segmentation models. (B) Fruit stripe classification models. (C) Flesh and cavity segmentation models. (D) Carpel number classification models. (E) Fruit shape classification models. (F) Fruit tumor detection models.

A U-Net semantic segmentation model was used for fruit stripe classification, and a VGG16 model was used as the trunk feature extraction network. A total of 1,811 samples were labeled. The model was trained with a cross-entropy loss function and the Adam optimization algorithm, which exhibited a maximum learning rate of 0.0001, a decreasing learning rate schedule of cos, a momentum factor of 0.9, 50 epochs, a batch size of 8, and pretraining weights. The loss declined quickly in the first 10 epochs (Fig. 3A), followed by minor fluctuations, which exhibited a general downward trend. Between the 30th and 35th epochs, the loss gradually stabilized and converged.

A ResNet50 classification model was used for stripe classification. The network’s input was the binary images obtained from the semantic segmentation process. Moreover, its output contained 5 stripe types (Fig. 2D), i.e., “small stripe at tip”, “continuous fragmented stripe”, “continuous connected stripe”, “continuous linear stripe”, and “no stripe”. The training dataset contained 1,608 images, including 270 images with small stripes at the tip, 193 images with continuous fragmented stripes, 382 images with continuous connected stripes, 221 images with continuous linear stripes, and 552 images with no stripes. Due to the small number of qualified samples and imbalances between different types, the training data need to be augmented to ensure the model training effectiveness. The augmentations included random rotation by 20°, random vertical flipping, random brightness enhancement ranging from 10% to 200%, and a random offset of 10% in height, which resulted in a total of 32,160 samples. The model was trained with 224 × 224 × 3 input images, an initial learning rate of 0.0002, a cross-entropy loss function, the Adam optimization algorithm, 300 epochs, a batch size of 16, and pretrained weights (Fig. 3B).

The U-Net model was used for performing endocarp segmentation, and a total of 1,169 samples were labeled. The training environment and profile were identical to those for stripe training. In the first 5 epochs, the loss decreased significantly. After 10 epochs, the loss reached its lowest point and then stabilized and converged (Fig. 3C). Similar to fruit segmentation, the pixel length in the green color card of the original image and the actual length were used to calculate the unit length (pixels per centimeter) for the image. This unit length, combined with the segmentation results from which the pixel areas of the sarcocarp and endocarp can be derived, enabled the calculation of parameters such as sarcocarp thickness, endocarp thickness, and the endocarp ratio.

A MobileNetV2 classification model was used for carpel classification. The input was the endocarp segmentation image, and there were 4 types of outputs (Fig. 2E), i.e., “2-carpel”, “3-carpel”, “4-carpel” and “5-carpel” types. The training dataset contained 1,369 images, including 45 images of 2-carpel type, 882 images of 3-carpel type, 381 images of 4-carpel type, and 61 images of 5-carpel type. After dividing the dataset into training and validation sets, the data augmentations were used to ensure that the different types of outputs had balanced numbers of samples, including random rotation by 60°, random offset of 10% in height, random offset of 10% in width, and random vertical flipping, which resulted in a total of 27,380 samples. The model profile was the same as that for the stripe classification model, with 300 training epochs and pretrained weights (Fig. 3D).

Furthermore, the MobileNetV2 classification model was used for fruit shape classification. The input data were black-and-white binary images of cucumber fruits, and the output data included 6 types (Fig. 2C), i.e., rods, cylinders, ellipses, inverted eggs, spindles, and heterotypes. The training dataset contained 1,744 images, including 565 images of rod type, 648 images of cylinder type, 119 images of ellipse type, 135 images of inverted-egg type, 135 images of spindle type, and 156 images of heterotype. After dividing the dataset into training and validation sets, data augmentations were used, including random rotations by 10°, random offsets of 20% in height, and random vertical flipping, which resulted in a total of 34,880 samples. The training profile was the same as above, with 300 training epochs and pretrained weights (Fig. 3E).

A YOLOv5 object identification model was used for tumor identification. The YOLOv5 model comprises a backbone network based on the cross-stage partial architecture, a neck of the feature pyramid network structure, and a detection head for feature extraction, feature enhancement, and feature point object prediction, respectively. Fruit tumors on the fruit were labeled using labelimg, and a total of 1,811 samples were labeled. The loss function for YOLOv5 comprised classification, localization, and confidence losses. The confidence and classification losses used binary cross entropy, while the localization loss used the complete intersection over union loss. The Adam optimizer was used for training. YOLOv5 was used as the model profile with an initial learning rate of 0.001, 200 epochs, and 30 early stopping rounds. During the first 10 rounds, the loss decreased drastically and then stabilized and converged (Fig. 3F).

### Algorithm validation

The metrics for analyzing various popular models for stripe segmentation and endocarp semantic segmentation, carpel classification, stripe classification, type classification, and tumor identification were compared to determine the best performing models.

The mean intersection over union (MIoU), mean pixel accuracy (MPA), and pixel accuracy (PA) of the U-Net model for stripe and endocarp segmentations were compared with those of the SegFormer, HRNet, and PSPNet models.

**Fig. 4.**
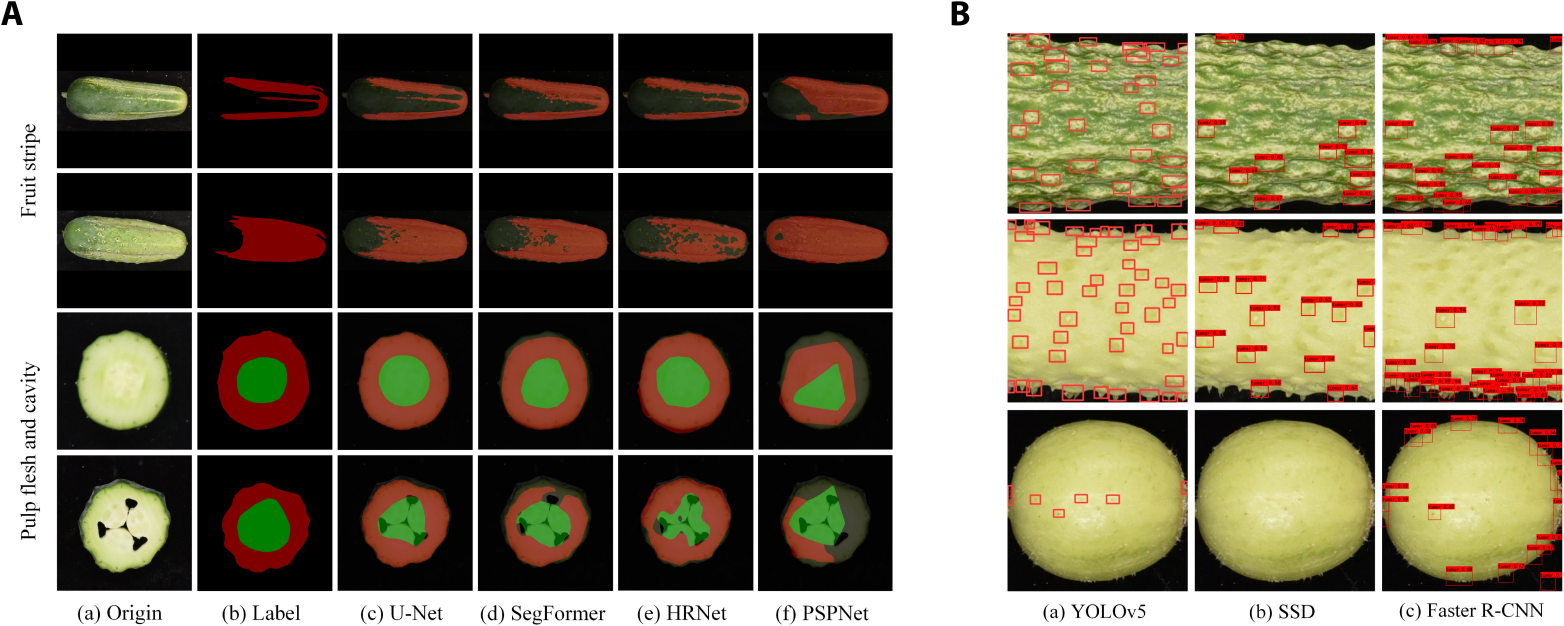
Comparison of image segmentation and fruit tumor detection. (A) Comparison of model segmentation images. (B) Comparison of model detection images.

The results (Table 2) show that for stripe segmentation, the U-Net model exhibited the highest MIoU (82.83%) and MPA (96.91%) values, while its MPA value of 91.86% was slightly lower than that of the PSPNet model (92.03%). The PSPNet model achieved the lowest values for the MIoU and PA, while the HRNet model achieved the lowest MPA (89.89%). For endocarp segmentation, the U-Net model achieved the highest values for MIoU (94.78%), MPA (97.23%), and PA (98.55%) among all the models, while the PSPNet model achieved the lowest values across all the indicators. This indicates that the U-Net model can achieve better segmentation in stripe and endocarp segmentations. A comparison of the segmentation images of these models with manual labels (Fig. 4A) revealed that segmentation with the U-Net model was the most accurate.

**Table 2.** Comparison of the segmentation model evaluation results

Models	Fruit stripe			Flesh and cavity		
	MioU (%)	MPA (%)	PA (%)	MioU (%)	MPA (%)	PA (%)
U-Net	82.83	91.86	96.91	94.78	97.23	98.55
SegFormer	79.22	91.8	95.9	90.66	94.71	97.23
HRNet	79.19	89.89	96.06	91.47	95.16	97.49
PSPNet	76.13	92.03	94.83	74.56	82.83	90.94

The accuracy, recall, and precision of the ResNet50 model for stripe classification, CarpelMobileNet for carpel classification, and ShapeMobileNet for shape classification were compared with those of other classification models. Among the models for stripe classification (Table 3), the ResNet50 model demonstrated the highest values for accuracy, recall, and precision, which were 97.41%, 97.39%, and 97.40%, respectively, followed by the VGG16 model, while the MobileNetV2 model achieved the lowest values of 94.71%, 94.65%, and 94.68%, respectively. Among the models for carpel counting, CarpelMobileNet obtained the highest values for accuracy, recall and precision, which were 97.85%, 97.89%, and 97.88%, respectively, followed by the ResNet50 model, which was slightly poorer than that of CarpelMobileNet, while the ViT-B/16 model achieved the lowest values of 92.49%, 91.90%, and 93.09%, respectively. Among the models for type classification (Table 4), ShapeMobileNet achieved the highest values for accuracy, recall and precision, which were 88.06%, 87.41%, and 87.73%, respectively, followed by the ViT-B/16 and VGG16 models, while the ResNet50 model achieved the lowest values of 83.33%, 82.73%, and 84.04%, respectively. The random forest model trained with feature parameters extracted through image recognition achieved an accuracy and precision of 85.11% and 86.59%, respectively, which are higher than the 84.44% and 84.53%, respectively, demonstrated by the VGG16 model. However, its recall value (62.86%) was the lowest and was considerably lower than that of the ResNet50 model (82.73%).

**Table 3.** Comparison of the classification model evaluation results

Models	Fruit stripe type			Flesh carpel number		
	Accuracy (%)	Recall (%)	Precision (%)	Accuracy (%)	Recall (%)	Precision (%)
VGG16	95.85	95.83	95.87	96.67	96.56	96.96
ResNet50	97.41	97.39	97.40	97.64	97.61	97.72
MobileNetv2	94.71	94.65	94.68	97.85	97.89	97.88
ViT-B/16	95.02	94.98	95.09	92.49	91.90	93.09

**Table 4.** Comparison of the fruit shape classification model evaluation results

Models	Fruit shape		
	Accuracy (%)	Recall (%)	Precision (%)
VGG16	84.44%	83.53%	84.53%
ResNet50	83.33%	82.73%	84.04%
MobileNetv2	88.06%	87.41%	87.73%
ViT-B/16	86.11%	85.38%	85.93%
Random forest	85.11%	62.86%	86.59%

The precision, recall, mAP@0.5, and mAP@0.5: 0.95 of the YOLOv5 object identification model for tumor identification were compared with those of the SSD and Faster R-CNN (Table 5). The YOLOv5 model achieved the highest values for precision, recall, mAP@0.5, and mAP@0.5: 0.95, which were 86.2%, 89.7%, 92.4%, and 40.1%, respectively. The SSD model achieved the lowest recall, mAP@0.5, and mAP@0.5: 0.95, which were 44.81%, 62.21%, and 16.2%, respectively, while the R-CNN model achieved the lowest precision value of 63.09%. Figure 4B shows that the performance of the YOLOv5 model was the best and most accurate among all the analyzed models.

**Table 5.** Comparison of detection model evaluation results

Models	Fruit tumor			
	Precision (%)	Recall (%)	mAP@0.5 (%)	mAP@0.5:0.95 (%)
YOLOv5	86.2	89.7	92.4	40.1
SSD	79.04	44.81	62.21	16.2
Faster R-CNN	63.09	84.29	80.4	25.3

### A correlation analysis of the traditional manually measured data and image-recognition-based data

**Fig. 5.**
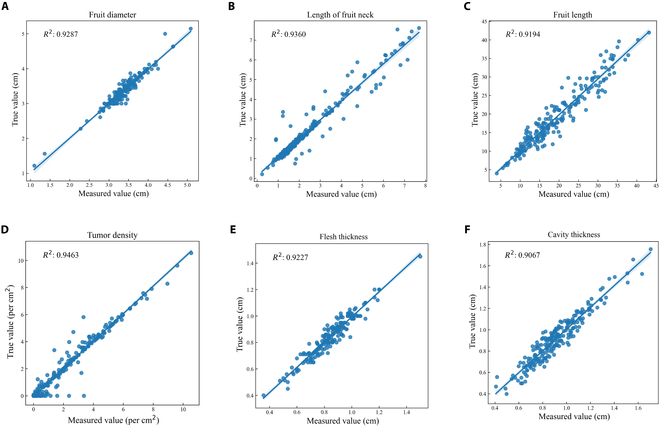
Analysis of the consistency between traditional measurements and image recognition shape parameters. (A to C) Correlation analysis of fruit diameter, length of fruit neck, and fruit length. (D to F) Correlation analysis of fruit tumor density, flesh thickness, and cavity thickness.

In this study, the fruit diameter, the length of the fruit neck, and the length of the entire fruit were measured by both traditional and image recognition methods. The *R*^2^ value of the fruit diameter data was 0.9287 (Fig. 5A), the *R*^2^ value of the neck length data was 0.936 (Fig. 5B), and the *R*^2^ value of the 2 sets of fruit length data was 0.9194 (Fig. 5C). The number of fruit tumors was also counted under a microscope and converted to tumor density. The 2 tumor density datasets were strongly correlated (*R*^2^ = 0.9463, Fig. 5D). We also compared the thickness data of the mesocarp and endocarp based on image segmentations with the actual Vernier caliper measurement data, and the *R*^2^ values reached 0.9227 and 0.9067, respectively (Fig. 5E and F). A correlation analysis showed that traditional measurements and image recognition data have high consistency.

The angle of the fruit neck and the angle of the fruit tip are important breeding traits for cucumbers. In previous research, we found that the traits related to fruit tips are quantitative traits, the indicators of which are normally distributed within a certain data range, such as the angle of the cucumber fruit tip within the range of 99.4° to 174.1° [11]. However, in traditional identification methods, the angle of the fruit tip is classified into 2 categories: thin and round.

In this study, a computer-assisted image recognition method was used to measure the tips and necks of cucumbers using 3 decomposition indicators, namely, angle, index, and proportion (Fig. 2A). The typical correlation analysis method was used to compare the grading data of the fruit neck and tip data with the image recognition data. The correlation between the 2 sets of data reached 0.847 (fruit neck) and 0.758 (fruit tip) (Table 6).

**Table 6.** Typical correlation analysis of fruit

Traits	Correlation	Eigenvalue	Wilks state	*F* value	Sig.
Fruit neck	0.847	2.532	0.283	189.883	−0
Fruit tip	0.758	1.347	0.426	100.596	−0
Fruit ribs	0.557	0.451	0.689	20.094	1.11 × 10^−16^
Fruit tumor	0.615	0.610	0.621	27.191	−0

### Smoothness of the fruit surface

**Fig. 6.**
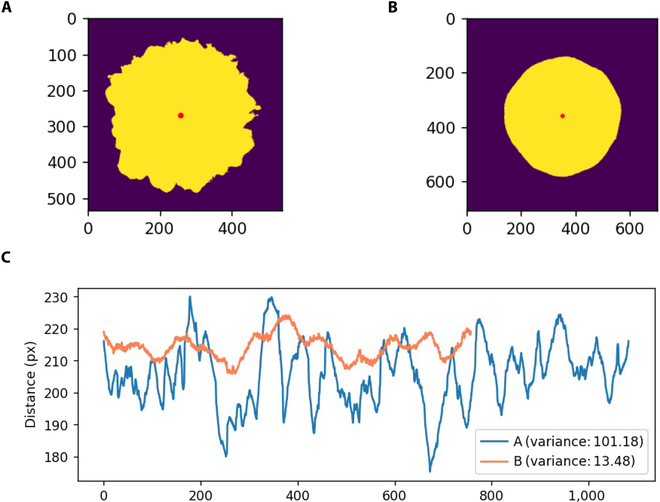
Analysis of surface smoothness. (A) Cucumber section segmentation with low smoothness. (B) Cucumber section segmentation with high smoothness. (C) Variance in the distance from the centroid to the edge in A and B.

The smoothness of the fruit surface directly affects the commercial properties of cucumber. In cucumber, smoothness is usually evaluated by the depth of the fruit rib and the size of the fruit tumor, which are traditional grading indicators. In the present image recognition method, 5 indicators, namely, *Hrs*, *Crs*, *Rls*, *Cfs*, and *Cdv*, were used to evaluate the degree of smoothness of cucumber fruits. Fruits with smooth surfaces were considered to have higher *Hrs*, *Crs*, *Rls*, and *Cfs* values as well as lower *Cdv* (Fig. 6A to C). A typical correlation analysis was performed between *Gfr* and *Gtu* with the above 5 image recognition-based indicators. The 2 groups of data were positive, and their correlation coefficients were 0.557 and 0.615, respectively (Table 6).

### Clustering of cucumber varieties using image recognition-based data

**Fig. 7.**
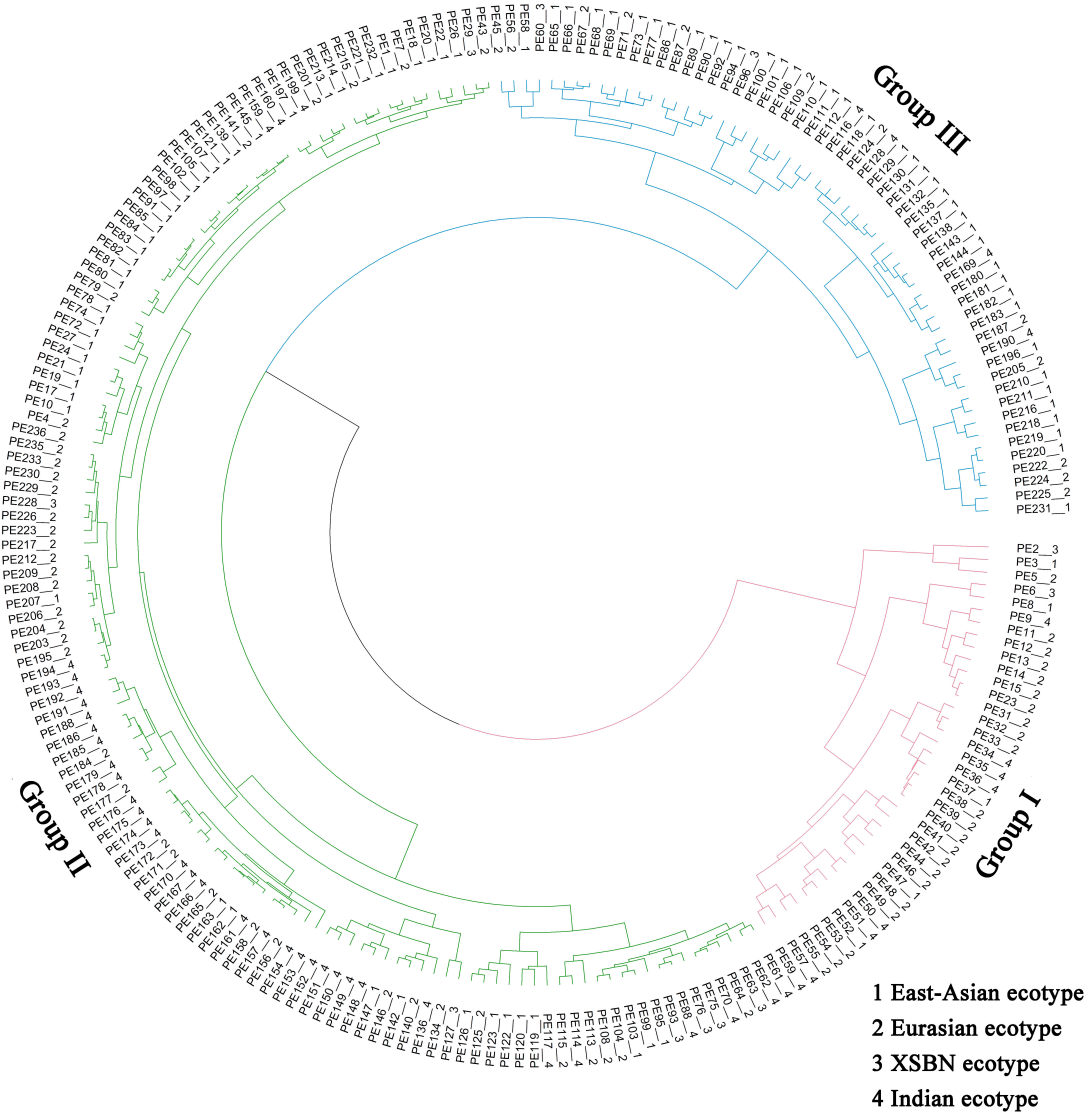
Cluster analysis of 229 cucumber inbred lines using image recognition-based data.

Cluster analysis of 229 cucumber inbred lines was performed based on the data collected by computer-assisted image recognition. The accession numbers of these cucumber lines refer to our previous study [39]. The image recognition data of 46 fruit traits were used for clustering, which showed a significant variability among the 229 cucumber accessions (Table S1). The cucumber population can be divided into 3 groups (Fig. 7). Thirty-seven varieties belonging to Group I, which were small in size, had short fruit lengths and diameters, and were primarily Eurasian and Indian ecotypes. Group II was the largest group, containing 134 cucumber varieties distributed across all 4 ecotypes with long but thin fruiting bodies and greater curvature (*Cv*). Group III included 58 varieties, which were characterized mainly by large fruit sizes and low fruit indices (*Fd*/*Fl*), mainly of the East Asian and Eurasia ecotypes.

### Implementation of CucumberAI

**Fig. 8.**
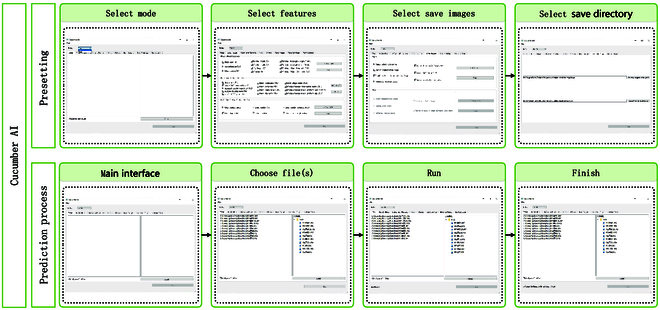
Data visualization and usage of the CucumberAI platform.

The cucumber feature analysis software CucumberAI was developed by combining image processing algorithms and deep learning models. CucumberAI was developed based on Python and has 2 main functions: fruit and flesh feature extraction. The results provide a diversified output display of XLS tables and detection process images. A guide for using CucumberAI is shown in Fig. 8. Fruit feature extraction has the following functions: (a) cucumber shape feature extraction, including the area, perimeter, volume, and length of each segment. (b) The identification of fruit tumors, including the number of densely packed areas of fruit tumors. (c) Cucumber stripe recognition, including the distribution and types of cucumber stripes and the proportion of cucumber stripes. The functions of fruit flesh feature extraction include (a) geometric feature extraction, including the circle filling rate, proportion of convex hull area, variance of centroid to contour distance, etc. and (b) extraction of flesh endocarp features, including flesh endocarp area, flesh endocarp thickness, etc. When using the software, the user first selects the running mode, fruit analysis or flesh analysis, then uploads the image, checks the features to be extracted, and finally clicks “Start”. When “Processing completed” appears, the analysis is completed, and the results are generated in the selected file folder.

## Discussion

In this study, we develop a new software, CucumberAI, for comprehensive, rapid, and accurate identification of cucumber traits and provide key support for the quantitative analysis and genetic breeding of cucumbers. The key advances underlying our model include (a) establishing a comprehensive list of cucumber fruit morphological traits, which includes 3 categories, fruit shape, fruit surface, and fruit flesh. There are 4 subcategories, shape of fruit contour, shape of fruit neck, shape of fruit tip, and smoothness, totaling 51 individual trait parameters. (b) Complex quantitative indicators for fruit tumors, stripe types, and endocarps were proposed for the first time, and a corresponding unique acquisition technology pipeline was established. (c) We propose a set of quantifiable smoothness metrics, considering that the smoothness of the fruit surface is crucial for cucumber pricing, and commonly used measurement indicators such as fruit ribs and fruit tumor size are extremely difficult to obtain. (d) In contrast to existing studies [17], we construct a detection algorithm architecture to accurately segment cucumber various fruit parts, which solved the problems of incorrect branching, twisting, and missing ends of the skeleton caused by burrs and distortions of cucumbers, and is crucial for detecting cucumber traits. (e) We built different types of cucumber image datasets to provide a foundation for broader research.

In this work, CucumberAI confronted some traits that were difficult to identify with traditional methods and demonstrated its strong detection capabilities. The algorithm integrates advanced deep learning algorithms to achieve stripe segmentation and classification, flesh endocarp segmentation, carpel classification, shape classification, and fruit tumor target detection, providing strong support for obtaining several difficult indicators, such as stripe type, fruit tumor density, carpel number, and shape classification. We conducted a series of benchmark tests to compare our results with those of advanced models. In the stripe segmentation and flesh endocarp segmentation experiments, the MIoU and MPA of the U-Net model for stripe segmentation were the highest at 82.83% and 91.86%, respectively, while the PA was 96.91%, similar to the best results. The U-Net model better preserves the details of the stripes and has better adaptability to complex stripe distributions. Other models are unable to effectively segment linear stripes and accurately identify large-scale distributed stripes. The MIoU, MPA, and PA of the U-Net model for fruit flesh endocarp segmentation were 94.78%, 97.23%, and 98.55%, respectively, which were the highest among all the models. The boundary of the segmented fruit flesh is clearer, and the segmented endocarp area is fuller. There is no situation where the fruit flesh area in other models exceeds the actual boundary or is missing, or the endocarp is too small. The visual segmentation effect of the model was similar to that of manual annotation, indicating that the U-Net model could effectively complete the stripe and flesh endocarp segmentation tasks.

The accuracies of the carpel classification model and stripe classification model were greater than 97%, and the accuracy of the type classification was greater than 88%. The precision and recall also achieved ideal results. The carpel classification results indicate that carpel counting can be achieved by classifying the features of the flesh endocarp. It is difficult to directly classify the shape of cucumber fruits into 11 types. Using a 3-stage combination of experience and AI classification models to distinguish fruit types can improve the classification accuracy. The precision, recall, mAP@0.5, and mAP@0.5:0.95 of YOLOv5 for fruit tumor detection were 86.2%, 89.7%, 92.4%, and 40.1%, respectively, greater than those of the other methods. The YOLOv5 model can effectively detect tumors in scenes where the color of the tumor is similar, or not similar, to the color of the fruit and can accurately detect tumors at the boundary. The overall performance is superior in general. Considering that computing resources are often scarce in agricultural applications, we adopt low-complexity deep learning models in CucumberAI with low equipment requirements and maintenance costs; these deep learning models are easy to deploy and apply locally and achieve ideal performances.

We further compared the *R*^2^ values of 6 numerical traits obtained from image recognition and manual measurements, which ranged between 0.9067 and 0.9463 (Fig. 5), indicating high correlations, demonstrating the reliability of computer-assisted recognition in measuring numerical indicators. In traditional measurements, complex traits such as the shape of the fruit neck and tip, rib depth, tumor size, tumor density, and stripes are usually defined by using a grading index that can meet the requirements for variety comparisons or postharvest product gradings, but they are not efficient in genetic mapping studies. Currently, only the fruit tumor gene *Tu*, the carpel number gene *CLV3* and the white peel gene *W* have been cloned using the grading method because these traits are controlled by a single gene [6,44,45]. In view of the limitations of the grading methods used in genetic research, we decomposed complex traits such as the shape of the fruit neck and fruit tip into 3 indices—angle, *D*: *R* index and proportion—and obtained correlations of 0.758 and 0.847, respectively, with the grading data. The smoothness of the fruit surface was divided into 5 indicators: *Hrs*, *Crs*, *Rls*, *Cfs*, and *Cdv*. The correlations also reached 0.557 and 0.615, respectively (Table 6). Although the correlation of complex trait data measured by image recognition and the manual methods was lower than that of the numerical indices mentioned above, we found that the data from image recognition had an obvious advantage in genetic mapping studies. For instance, in our previous study, 2 major effect loci controlling the shape of fruit tips were detected by using grading data for fruit tips [11]. After recalculating the image recognition data, we detected 2 new genetic loci in addition to a significantly narrowed interval between the major loci (unpublished data), suggesting that CucumberAI has great potential for revealing the genetic basis of complex traits in cucumber fruits.

Currently, CucumberAI still has several limitations, such as the identification of stripe traits, for which we still use a grading index (Fig. 2D). Additionally, fruit color has been explored by other image recognition methods, which have shown accurate results in the recognition of local colors. However, due to the influence of surface structures, such as the stripes, tumors, and spines, the numerical indicators that can define the overall fruit color have not yet been identified.

CucumberAI is the first complete pipeline for determining cucumber fruit morphological traits, and 32 parameters were adopted for the first time. From the resulting measures, it is possible to extract information on a variety of characteristics. CucumberAI is faster and easier to use than the manual methods while producing similar results. Moreover, the image recognition strategy replaces the traditional method of trait grading, and all the traits are described in a data-driven manner. For complex traits, the results are more accurate. There are still existing challenges and difficulties, including the need to collect more samples of cucumber shapes and color traits. We will continue to track the latest progress in segmentation, classification, and object detection in the field of AI and continuously update the models. CucumberAI aims to promote cucumber fruit breeding efficiency and advance the development of fruit models. In the future, more samples will be collected to expand the data on cucumber species and to optimize the model parameters. In the Cucurbitaceae family, the formation of gourd fruits follows similar patterns, especially since cucumbers and melons both belong to the genus *Cucumis*, the fruits of which exhibit a strong resemblance in appearance. Therefore, the software developed in this study can be fully applied for accurate identification and genetic breeding of melon fruit. Moreover, although the diversity of fruit-bearing crops in nature is very high, basic fruit architecture encompasses the proliferation, growth, and arrangement of cells, which follow similar patterns. Therefore, the strategies for decomposing fruit appearance traits and the core algorithms for image recognition proposed in this project can be applied to other fruit-bearing crops.

## Data Availability

All data underlying this study are available in the Cucumber dataset (https: //zenodo.org/records/10081197) at Zenodo. The software used for image analysis in this work includes PC and web versions. The PC version is available in Cucumber (https://zenodo.org/records/10039037) at Zenodo, and the web version can be accessed through the website http://47.100.80.240:31/.

## Supplementary Materials

Supplementary 1 Table S1. Range (R) and coefficient of variation (CV = (SD/Mean) × 100%) of the 46 traits between the 229 cucumber accessions.
